# Prognostic value of LRRC4C in Colon and Gastric Cancers correlates with Tumour Microenvironment Immunity

**DOI:** 10.7150/ijbs.58876

**Published:** 2021-04-03

**Authors:** XiaoFeng Yang, Purun Lei, Lijun Huang, Xiao Tang, Bo Wei, HongBo Wei

**Affiliations:** Department of Gastrointestinal Surgery, The Third Affiliated Hospital of Sun Yat-sen University, Tianhe Road 600, Guangzhou 510630, China.

**Keywords:** TME, LRRC4C, ESTIMATE, CIBERSORT, gastric cancer, colon cancer

## Abstract

In this study, we aimed to use ESTIMATE and CIBERSORT computational methods to analyse transcriptional information on COAD and STAD in TCGA. We downloaded transcriptome RNA-seq data of 446 patients with colon cancer from TCGA and estimated the amount of immune and stromal components in the COAD samples using CIBERSORT algorithms. We analysed differentially expressed genes in 446 TCGA samples and 585 Series GSE39582 samples, in high- and low-scoring groups, using Cox regression. The expression of *LRRC4C*, correlated positively with clinicopathological characteristics and negatively with the survival of patients with COAD. Single-gene survival analysis using Gene Expression Profiling Interactive Analysis 2.0 and Kaplan-Meier plotter revealed an association between high levels of *LRRC4C* expression and poor prognosis in patients with colon and gastric cancers. Gene set enrichment analysis of COAD and STAD samples indicated that genes in groups expressing high and low *LRRC4C* levels were mainly enriched in immune-related activities and metabolic pathways, respectively. Difference and correlation analyses of the relationship between *LRRC4C* expression and tumour-infiltrating immune cells, determined using CIBERSORT algorithms, revealed that monocytes, resting mast cells, and M2 macrophages were positively correlated with *LRRC4C* expression.

## Introduction

Gastrointestinal cancers include gastric, colon, and oesophageal cancers and are the most extensive and prevalent type of malignancies worldwide. The incidence and mortality of colon and gastric cancers are among the top five globally 1. Cancer is presently classified according to the American Joint Committee on Cancer/Union for International Cancer Control guidelines that strictly rely on tumour characteristics, such as the extent of the primary tumour (T), the involvement of regional lymph nodes (N), and the presence of distant metastases (M) (TNM staging). This classification is one of the main tools for the routine prognostication and treatment of colon and gastric cancers 2. However, TNM staging does not provide the complete prognostic information beyond the tumour cells nor insights into the tumour immune status. Therefore, it might not predict responses to various therapeutic modalities.

Solid malignant colon and gastric tumours are composed of cancer cells and a tumour microenvironment (TME) comprising tumour-associated stromal cells [such as tumour-infiltrating immune cells (TICs), cancer-associated fibroblasts and endothelial cells], the extracellular matrix, and numerous metabolites and cytokines 3. Recently, attention is being focused on the importance of the TME in tumour development. Stromal cells play an important role in tumour growth, progression, metastasis, and drug resistance 4, 5. Immune cells engage the TME from the initiation of tumorigenesis. Tumours evolve through phases that profoundly impact the recruitment and phenotypes of TICs. The major TIC population includes tumour-associated macrophages (TAMs), which are commonly educated by tumour cells to become partners in crime, promoting tumour immune escape, angiogenesis, growth, and metastasis 6. Wang et al. demonstrated an abundant infiltration of FOXP3+ Treg cells in the gastric cancer tissue, which may predict a better prognosis of gastric cancer 7. The macrophage-specific deletion of hypoxia-inducible factor 2α reduces tumour infiltration by TAMs and improves the outcome of colitis-associated colon cancer 8. TICs are under metabolic stress as the tumour cells are characterized by abnormal metabolic activity, which may induce an impaired immune response, allowing tumour cells to evade the host immune system 9. The TME can activate or restrain tumour progression, malignancy, or metastasis and is used to assess cancer prognosis 5, 10.

The Estimation of STromal and Immune cells in MAlignant Tumor tissues using Expression (ESTIMATE) data is a tool that uses gene expression data for the prediction of tumour purity and the presence of infiltrating stromal/immune cells in the tumour tissues 11. Newman et al. developed the analytical tool Cell-type Identification By Estimating Relative Subsets Of known RNA Transcripts (CIBERSORT) to estimate the abundance of cell types in a mixed cell population using gene expression data 12. In this study, we aimed to use the ESTIMATE and CIBERSORT computational methods to analyse transcriptional information on colon adenocarcinoma (COAD) and stomach adenocarcinoma (STAD) using data from The Cancer Genome Atlas (TCGA). We calculated proportions of the TICs and ratios of the immune and stromal components in COAD and STAD samples to identify the predictive biomarker leucine-rich repeat containing 4C (*LRRC4C*; netrin-G1 ligand). This protein belongs to the LRR superfamily that also includes netrin-G2 ligand/LRRC4, and netrin-G3 ligand/LRRC4B 13. Although *LRRC4C* itself does not seem to be associated with cancer progression, *LRRC4/NGL-2* has been identified as a tumour suppressor gene for glioma and epithelial ovarian cancer 14, 15. Here, we analysed differentially expressed genes (DEGs) identified through comparisons between the immune and stromal components in COAD and STAD samples and found that *LRRC4C* might be an indicator of altered TME status in COAD and STAD.

## Materials and Methods

### Data preparation and estimation of stromal and immune scores

Transcriptome RNA-seq data of 446 patients with COAD and 348 patients with STAD (with survival information and expression data) and their corresponding clinical data were downloaded from TCGA (https://portal.gdc.cancer.gov/) at level 3. An independent dataset from the Gene Expression Omnibus (GEO) database was used for external validation and included 585 patients with colon cancer from Series GSE39582 16. Institutional review board approval and informed consent were not required to analyse the innominate data from these databases. The ESTIMATE algorithm was applied to estimate the ratios of the immune-stromal components in the TME of all samples, which were presented as immune, stromal, and ESTIMATE scores.

### Correlations between clinical characteristics and stromal/immune scores

Correlations between clinical characteristics and stromal/immune scores were analysed using SPSS 25.0. Student's t-test or one-way analysis of variance (ANOVA) was used to test significance depending on the number of clinical characteristics compared.

### Correlations between prognosis and stromal/immune scores

To achieve statistical significance and avoid arbitrary cut-point selection, we used X-tile software to select the optimal cut-off value of the stromal or immune scores of the colon cancer samples^17^, and the patients were divided into a low score group or a high-score group respectively. Then, survival was analysed using R software loaded with the survival and survminer packages. Kaplan-Meier survival curves were plotted; p < 0.05 (log-rank test) was considered statistically significant.

### Identification of DEGs

The 446 colon cancer samples had been divided into high or low stromal (or immune) groups based on the optimal cut-off score described above. The limma package was used to analyse differential gene expression, and DEGs were generated by a comparison between the two groups 18. A false discovery rate adjusted p < 0.0001, combined with a simultaneous absolute value of fold change |FC| ≥ 3, was set as the threshold for DEG identification.

### Enrichment analysis and construction of Protein-Protein Interaction (PPI) Network

Enrichment analysis of Gene Ontology terms, including biological process, cellular component, and molecular function, and that of the KEGG pathway were conducted for all DEGs shared in the stromal and immune groups using the Database for Annotation, Visualization and Integrated Discovery 19. A false discovery rate < 0.05 was set as the cut-off. The PPI network was retrieved from the STRING database 20 and reconstructed using Cytoscape 3.7.2 21.

### Cox regression analysis

The 446 patients with colon cancer from TCGA and the 585 patients from Series GSE39582 were divided into high- or low-expression groups for each DEG based on the median. R language loaded with survival and survminer packages was applied for the survival analysis. Kaplan-Meier method was used to plot the survival curve, and log-rank test was used as the statistical significance test; p < 0.05 was considered significant.

### TIMER 2.0 database analysis

The levels of *LRRC4C* expression in various tumours were analysed using the TIMER2.0 database (http://timer.cistrome.org/) 22. TIMER 2.0 applies a previously published statistical deconvolution method to infer the abundance of TICs from gene expression profiles. The TIMER2.0 database includes 10,897 samples across 32 cancer types from TCGA to estimate the abundance of immune infiltrates. We analysed *LRRC4C* expression in different types of cancer.

### Kaplan-Meier plotter database analysis

The Kaplan-Meier plotter (http://kmplot.com/analysis/) offers a means of readily exploring the impact of a wide array of genes on patient survival in 21 different types of cancer, with large sample sizes for breast (n = 6,234), ovarian (n = 2,190), lung (n = 3,452), and gastric (n = 1,440) cancer cohorts 23. Therefore, the associations between *LRRC4C* expression and prognosis in patients with breast (n = 5,353), ovarian (n = 3,091), lung (n = 2,909), and gastric (n = 1,517) cancers were analysed using the Kaplan-Meier plotter. Values with p < 0.05 were considered statistically significant.

### Survival analysis using GEPIA 2.0

GEPIA 2.0 is an online database that facilitates the standardized analysis of RNA-seq data from 9,736 cancer samples and 8,587 normal control samples in the TCGA and GTEx data sets (http://gepia2.cancer-pku.cn/#index) 24. We, therefore, employed this database to assess the link between *LRRC4C* expression and patient prognosis in multiple cancer types and drew the survival curve plot between them.

### Gene Set Enrichment Analysis (GSEA)

Hallmark and C7 collections were downloaded from the Molecular Signatures Database as target sets for GSEA using the GSEA v3.0 software downloaded from The Broad Institute. Whole transcriptomes of all tumour samples were assessed using GSEA, and only gene sets with nominal p < 0.05 and a false discovery rate of q < 0.05 were considered significant.

### TIC profiles

**Figure 1:**
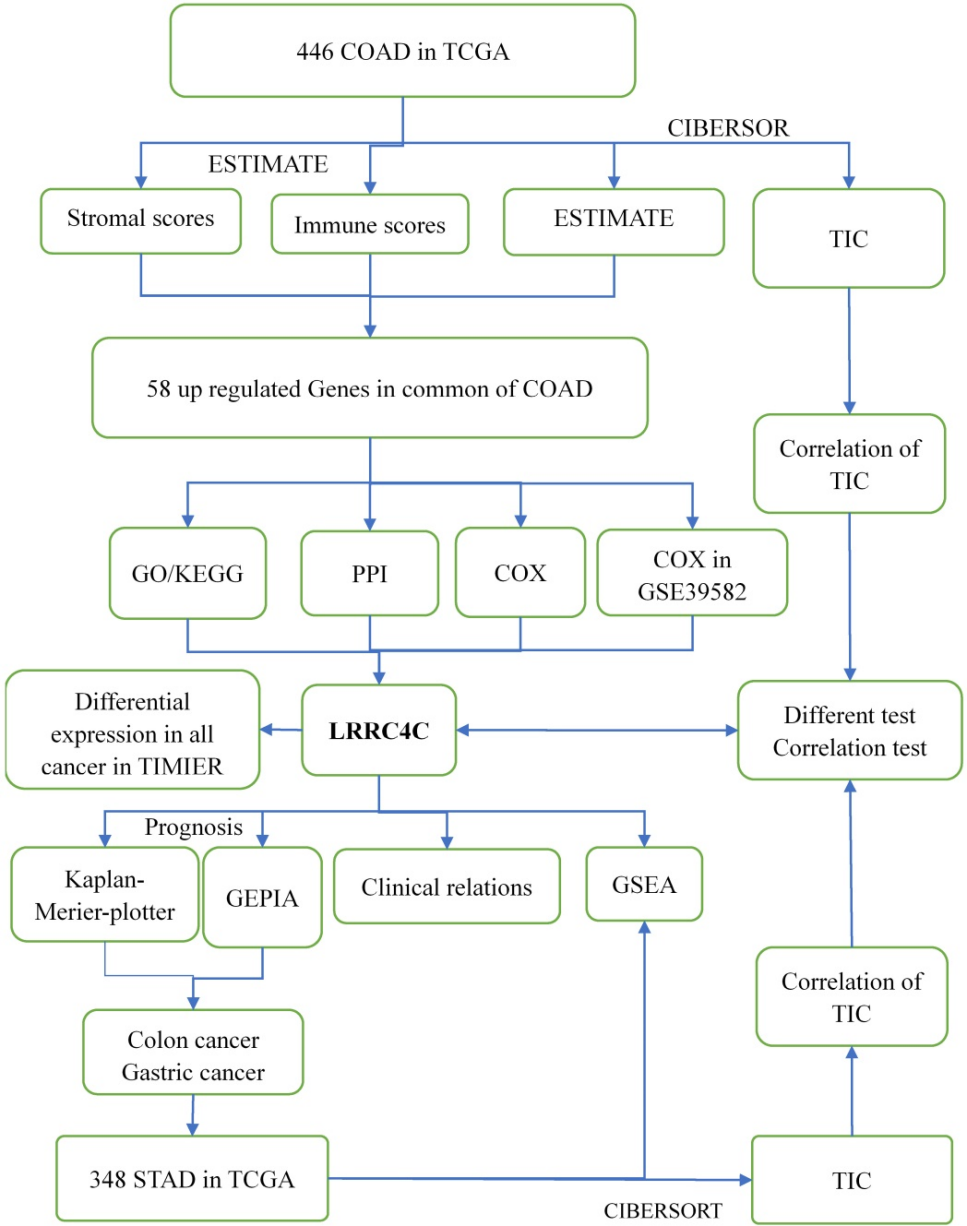
Flow diagram of this study

We estimated the TIC abundance in all gastric and colon cancer samples using CIBERSORT and selected TICs with p < 0.05 using quality filtering. Figure 1 summarizes the analytical process applied in the present study.

## Results

### Associations of stromal and immune scores with colon cancer features and prognosis

**Figure 2:**
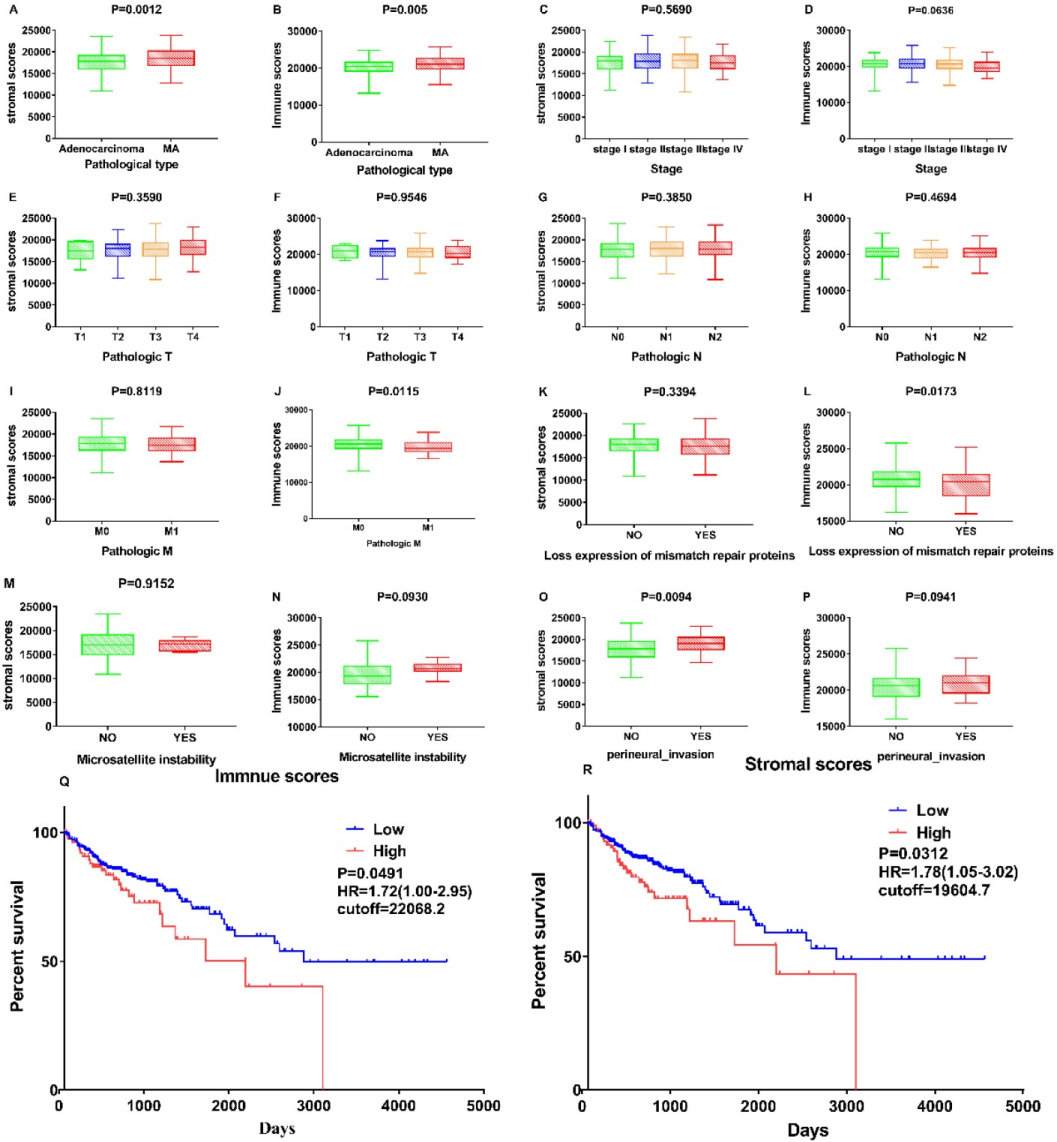
** Correlation of Stromal Scores and Immune Scores with clinicopathological characteristics. (A, B)** Distribution of Stromal Scores and Immune Scores in pathological type. The P = 0.0012 and 0.005, respectively, by Student's test. **(C-D)** Distribution of Stromal Scores and Immune Scores in stage. The p = 0.5690 and 0.0636, respectively, by One-Way ANOVA test. (E-F) Distribution of scores in T classification. The p = 0.3590 and 0.9546, respectively, by One-Way ANOVA test. **(G-H)** Distribution of scores in N classification. The p = 0.3850 and 0.4694, respectively, by Student's test. **(I-J)** Distribution of scores in M classification. The p = 0.8119 and 0.0115, respectively, by Student's test. (K-L) Distribution of scores in expression of mismatch repair. The p = 0.8119 and 0.0115, respectively, by Student's test. **(M-N)** Distribution of scores in Microsatellite instability. The p = 0.9152 and 0.0930, respectively, by Student's test. **(O-P)** Distribution of scores in perineural invasion. The p = 0.0094 and 0.0941, respectively, by Student's test. **(Q)** Kaplan-Meier survival analysis for COAD patients grouped into high or low score in Immune Scores determined by the comparison with the cutoff=22068.2. p = 0.0491, HR=1.72(1.00-2.95) by log-rank test. **(R)** Kaplan-Meier survival curve for Stromal Scores with p = 0.0312, HR=1.78(1.05-3.02) by log-rank test (cutoff=19604.7).

Associations between stromal and immune scores and the clinical characteristics of patients with colon cancer were examined by comparing score distributions among histology classifications; TNM stages; and the status of mismatch repair proteins, microsatellite instability, and perineural invasion (Figure 2A-P). Stromal and immune scores were higher in mucous adenocarcinoma than in adenocarcinoma (Figure 2A and B; p = 0.0012 and p = 0.005, respectively; Student's t-test). Stromal and immune scores did not significantly differ with respect to tumour stage (p = 0.569 and p = 0.0636, respectively; one-way ANOVA). Immune scores correlated negatively with the metastases classification of TNM stages and the status of mismatch repair proteins (Figure 2J and L; p = 0.0115 and p = 0.0173, respectively; Student's t-test). Stromal scores correlated positively with perineural invasion status (Figure 2O; p = 0.0094; Student's t-test).

The association of stromal and immune scores with colon cancer prognosis was evaluated by dividing the patients into two groups based on these scores using standardized log-rank statistics (see Materials and Methods for details). The overall survival was better for patients with low, rather than high, stromal or immune scores (p = 0.0312, HR=1.78 (1.05-3.02) and p = 0.0491, HR=1.72 (1.00-2.95), respectively; log-rank tests) as indicated in Figure 2 Q-R. These results suggest that the ratio of immune and stromal components is associated with the progress of COAD, particularly with perineural invasion and metastasis, and could indicate the prognosis in these patients.

### Comparison of gene expression profiles by immune and stromal scores in colon cancer

**Figure 3:**
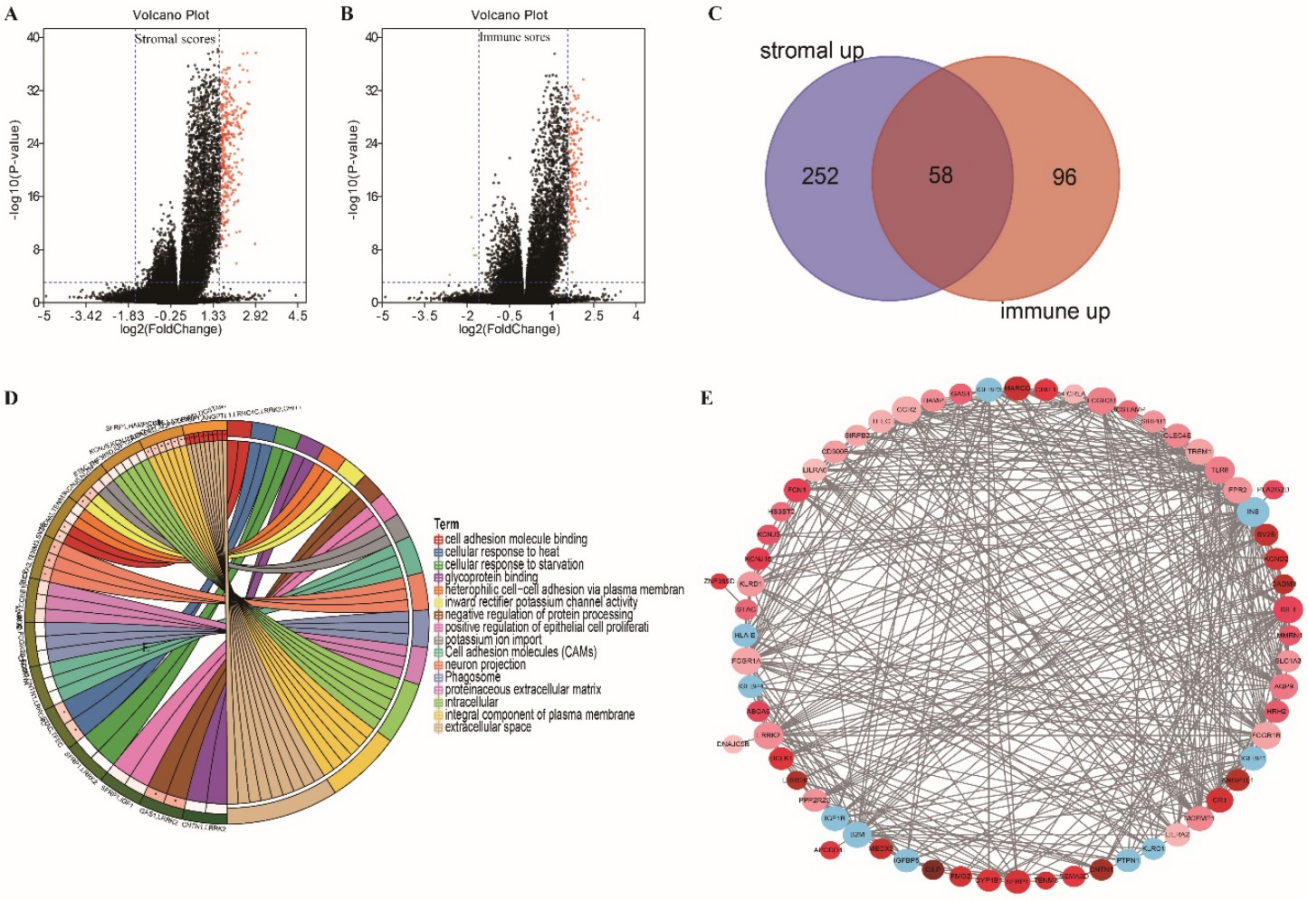
** Volcano plot, Venn plots, circle enrich plot, PPI network. (A-B)** Volcano for DEGs generated by comparison of the high score group vs the low score group in Stromal scores and Immune Scores. Differentially expressed genes were determined by Wilcoxon rank sum test with adjust. p-value< 0.001 and fold-change ≥3 after log2transformation as the significance threshold. **(C)** Venn plots showing common up-regulated and down-regulated DEGs shared by Immune Scores and Stromal Scores. **(D)** circle enrich plot. GO and KEGG enrichment analysis for 58 DEGs, terms with p and q < 0.05 were believed to be enriched significantly. **(E)** Interaction network constructed with the nodes with interaction confidence value >0.40.

Gene expression profiles in colon cancer were compared between patients with high or low stromal (or immune) scores to identify stromal (or immune) score-related DEGs. We identified 312 and 159 DEGs related to the stromal and immune scores, respectively (Figure 3A and B; |FC| ≥ 3, adjusted p < 0.0001). Among them, 310 and 154 DEGs were upregulated in high stromal and high immune scores, respectively. An intersection analysis displayed as a Venn diagram demonstrated that 58 upregulated DEGs were associated with high immune and stromal scores and that no downregulated genes were associated with low scores (Figure 3C). The results of biological function enrichment analyses demonstrated that the upregulated DEGs were mapped mostly to immune-related activities, such as cell adhesion binding, cellular responses to heat, and glycoprotein binding. These findings suggest that immune factor involvement is a predominant feature of the TME in COAD.

### Identification of prognostic DEGs in colon cancer

**Figure 4:**
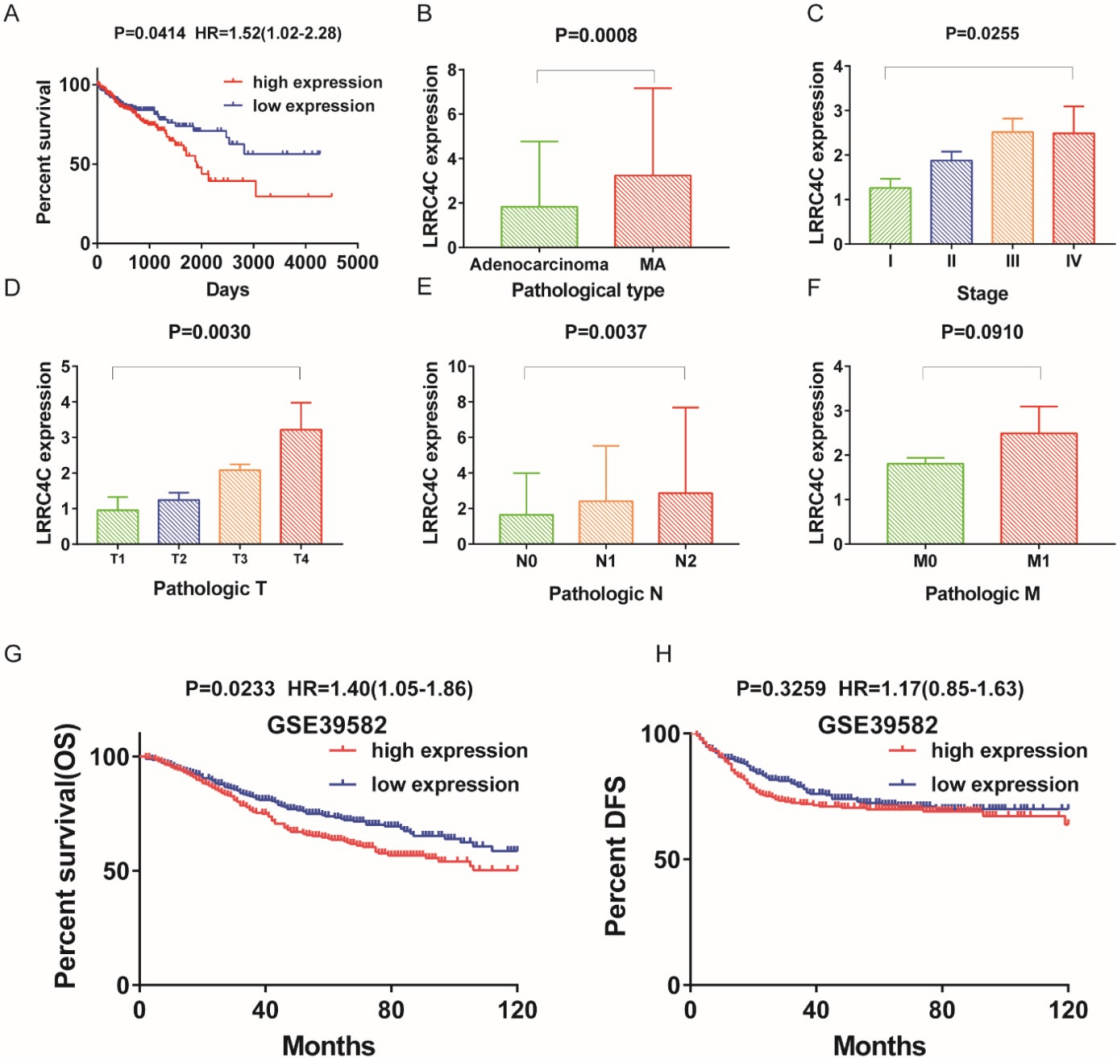
The differentiated expression of LRRC4C in samples and correlation with survival and clinicopathological characteristics of COAD patients. (A) Survival analysis for COAD patients with different LRRC4C expression. Patients were labeled with high expression or low expression depending on the comparison with the median expression level. p = 0.0414, HR=1.52(1.02-2.28) by log-rank test. (B) Differentiated expression of LRRC4C in the mucinous adenocarcinoma and adenocarcinoma sample, p=0.0008, by student's test. (C) The correlation of LRRC4C expression with clinicopathological staging Characteristics, P=0.0255, by One-Way ANOVA test. (D) Distribution of LRRC4C in T classification. The p = 0.0030, by One-Way ANOVA test. (E) Distribution of LRRC4C in N classification. The p = 0.0037, by One-Way ANOVA test. (F) Distribution of LRRC4C in M classification. The p = 0.0030, by Student's test. (G, H); Kaplan-Meier survival analysis for colon cancer patients in GSE39582 grouped into high or low LRRC4C expression determined by the comparison with the median. P = 0.0233, HR=1.40(1.05-1.86), by log-rank test for overall survival; P = 0.3295, HR=1.17(0.85-1.63) by log-rank test for DFS.

We investigated significant factors among 58 DEGs using univariate Cox regression analysis for survival of patients with colon cancer in the TCGA and GEO databases. Only *LRRC4C* was found to be a significant prognostic factor in both databases (Table S1, Figure 4A, G and H). These results suggested that high *LRRC4C* expression is associated with the poor prognosis of colon cancer.

### Correlations between *LRRC4C* expression and colon cancer features

We analysed *LRRC4C* expression combined with clinical characteristics (Figure 4B-F). The results of one-way ANOVA demonstrated a positive correlation between *LRRC4C* expression and TNM stages in patients with COAD (Figure 4C-F, p = 0.0255, p = 0.003, p = 0.0037 and p = 0.091 for TNM, T, N, and M stages, respectively). Similarly, increased *LRRC4C* expression was observed in colon mucinous adenocarcinoma samples compared with adenocarcinoma samples (Figure 4B; p = 0.0008; Student's t-test). These findings indicate that *LRRC4C* expression in the TME positively correlates with colon cancer progression.

### Expression of *LRRC4C* in various types of tumours and its prognostic value in cancer

To explore the relationship between *LRRC4C* and different types of tumours, we examined* LRRC4C* expression in various human cancers using RNA-seq data from the TIMER2.0 database. Figure S1 gives the levels of *LRRC4C* expression in tumours and matched normal tissues in all TCGA datasets. The expression of *LRRC4C* was significantly lower in colon, gastric, glioblastoma multiforme, and breast cancer tissues than in normal tissues. These results confirm the downregulation of the *LRRC4C* gene in various cancers compared with normal tissues.

**Figure 5:**
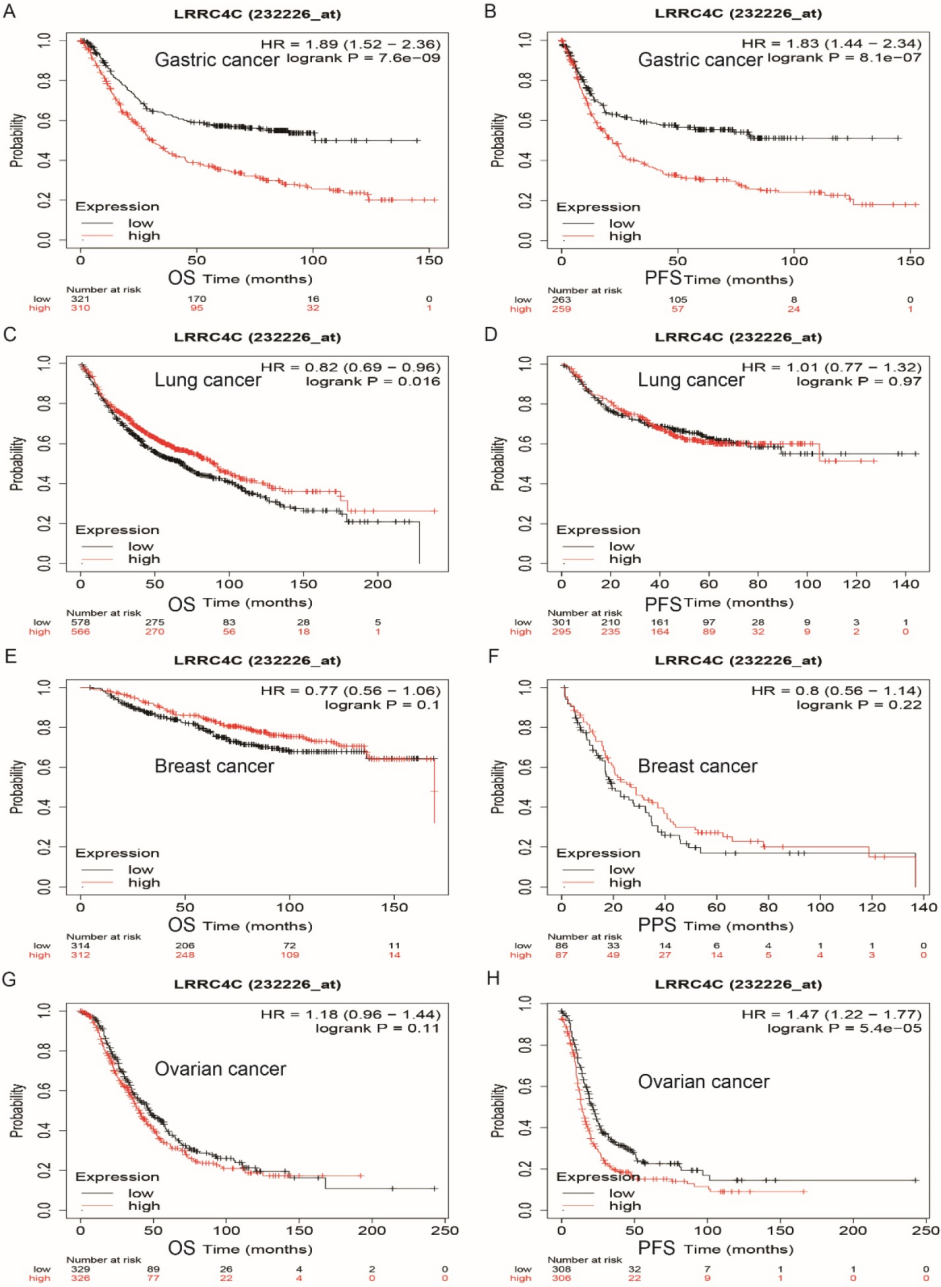
Survival curves of high or low expression of LRRC4C in different tumors from Kaplan-Meier plotter (A, B) OS and PFS survival curves of STAD (log-rank test, p=7.6e-09, HR=1.89(1.52-2.36) for OS and p=81e-07, HR=1.83(1.44-2.34) for PFS). (C, D) OS and PFS survival curves of lung cancer (log-rank test, p=0.016, HR=0.82(0.69-0.96) for OS and p=0.97, HR=1.01(0.77-1.32) for PFS). (E, F) OS and PPS survival curves of breast cancer (log-rank test, p=0.1, HR=0.77(0.56-1.06) for OS and p=0.22, HR=0.8(0.56-1.14) for PPS). (G, H) OS and PFS survival curves of ovarian cancer (log-rank test, p=0.11, HR=1.18(0.96-1.44) for OS and p=5.4e-05, HR=1.47(1.22-1.77) for PFS). OS, overall survival; PFS, progression-free survival; DFS, disease-free survival; HR: hazard ratio.

We explored the prognostic value of *LRRC4C* in human cancers using the Kaplan-Meier plotter, based on Affymetrix microarray data. Low levels of *LRRC4C* expression notably indicated better overall survival (OS) (hazard ratio [HR]: 1.89; 95% confidence interval [CI]: 1.52-2.36; p = 7.6e-09) and progression-free survival (PFS) (HR: 1.83; 95% CI: 1.4-2.34; p = 8.1e-07) compared with high levels of *LRRC4C* expression in gastric and colon cancers (Figures 5A and 5B). Similarly, low levels of *LRRC4C* expression indicated better PFS (HR: 1.47; 95% CI: 1.22-1.77, p = 5.4e-05) for ovarian cancer (Figure 5H). However, the prognosis in patients with lung cancer expressing low levels of *LRRC4C* was poor (Figure 5C; HR: 1.89; 95% CI: 1.52-2.36; p = 7.6e-09). Furthermore, *LRRC4C* expression was not associated with the prognosis of breast cancer.

**Figure 6:**
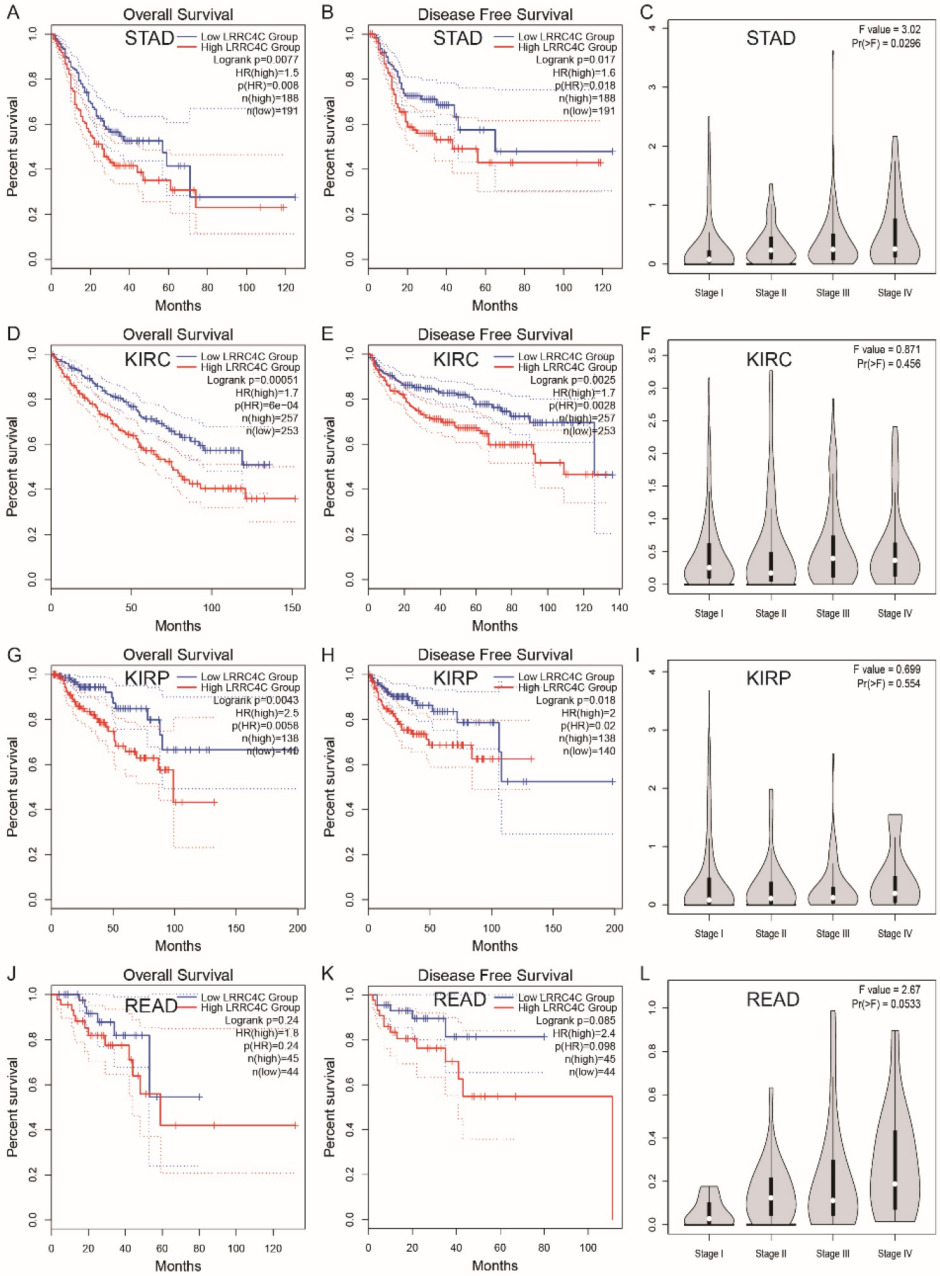
Survival curves of high or low expression of LRRC4C and the correlation of LRRC4C expression with clinicopathological staging Characteristics in different tumors from the GEPIA 2.0 database. (A, B) High LRRC4C expression was correlated with better OS and DFS than low LRRC4C expression in the STAD (n=379, log-rank test, p=0.0077, HR=1.5 for OS and p=0.017, HR=1.6 for PFS). (C) The correlation of LRRC4C expression with clinicopathological staging Characteristics in STAD, p=0.0296. (D, E) High LRRC4C expression was correlated with better OS and DFS than low LRRC4C expression in the KIRC (n=510, log-rank test, p=0.00051, HR=1.7 for OS and p=0.0025 HR=1.7 for PFS). (F) The correlation of LRRC4C expression with clinicopathological staging Characteristics in KIRC, p=0.456. (G, H) High LRRC4C expression was correlated with better OS and DFS than low LRRC4C expression in the KIRP (n=278, log-rank test, p=0.0043, HR=2.5 for OS and p=0.018, HR=2 for PFS). (I) The correlation of LRRC4C expression with clinicopathological staging Characteristics in KIRP, p=0.554. (J, K) High LRRC4C expression was correlated with better OS and DFS than low LRRC4C expression in the READ without statistic difference (n=99, log-rank test, p=0.24, HR=1.8 for OS and p=0.085, HR=2.4 for PFS). (L) The correlation of LRRC4C expression with clinicopathological staging Characteristics in READ, p=0.0533.

We also examined the prognostic value of *LRRC4C* for various tumours using RNA-seq data from TCGA and GEPIA 2.0 databases. The relationship between *LRRC4C* expression and survival in 33 cancer types was analysed (Figure S2). Compared with high expression, low expression of *LRRC4C* was associated with better OS or disease-free survival (DFS) in STAD (HR: 1.5; p = 0.0077 and HR: 1.6; p = 0.0017, respectively), kidney renal clear cell carcinoma (HR: 1.7; p = 0.00051 and HR: 1.7; p = 0.0025, respectively), and kidney renal papillary cell carcinoma (HR: 2.5; p = 0.0043 and HR: 2.0; p = 0.0018, respectively) (Figure 6A, B, D, E, G and H). Low *LRRC4C* expression was also associated with better OS or DFS in rectal adenocarcinoma (READ); however, the values were not statistically significant (Figure 6F). On exploring the relationship between *LRRC4C* expression and TNM stages, we found a significant positive correlation in patients with STAD (p = 0.0296; Figure 6C) and a positive but not significant correlation in patients with READ (p = 0.0533; Figure 6L). These results indicate the important prognostic value of *LRRC4C* in patients with gastric and colon cancers.

### GSEA

**Figure 7:**
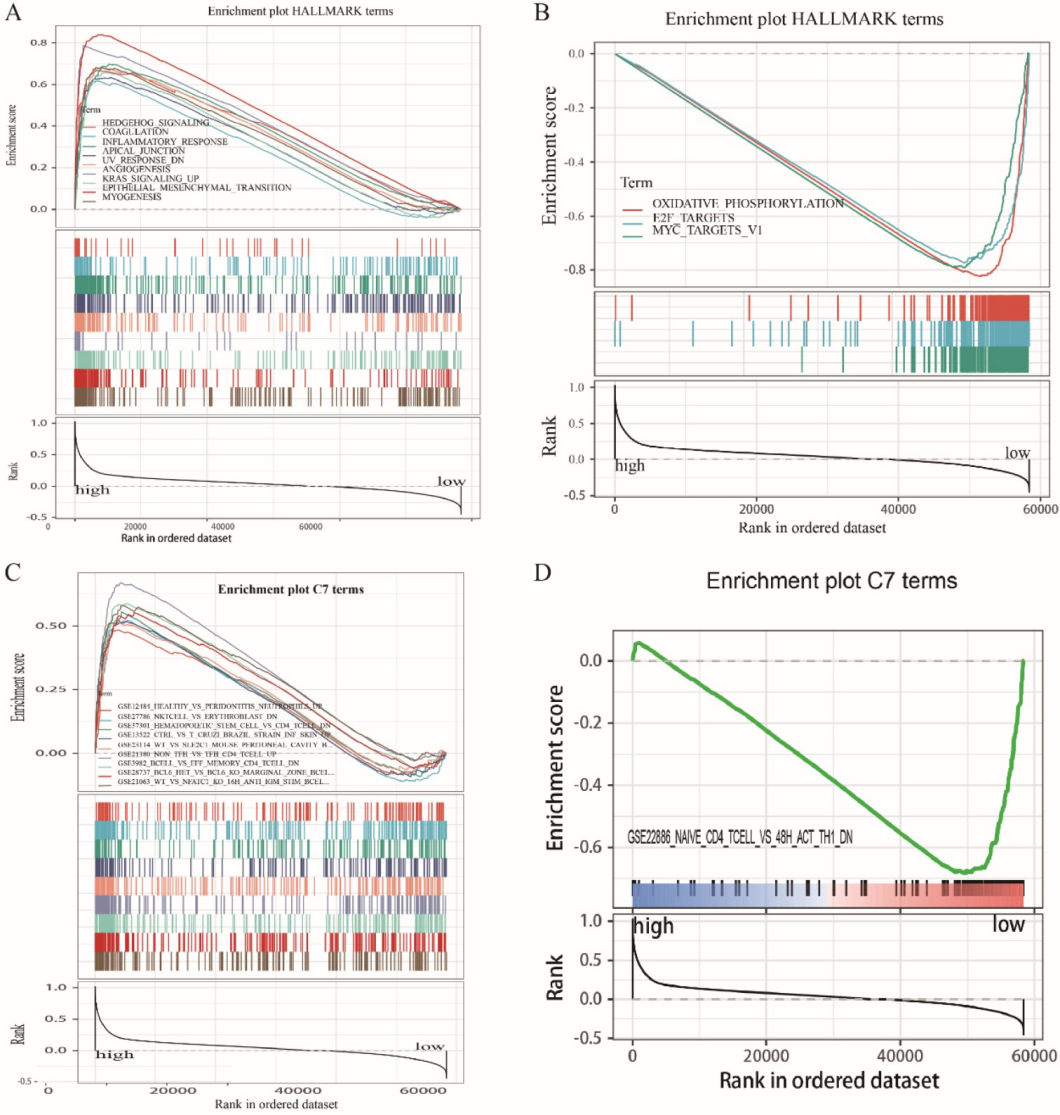
GSEA for COAD samples with high LRRC4C expression and low expression. (A) The enriched gene sets in HALLMARK collection by the high LRRC4C expression sample. Each line representing one particular gene set with unique color, and up-regulated genes located in the left approaching the origin of the coordinates, by contrast the down-regulated lay on the right of x-axis. Only gene sets with NOM p < 0.05 and FDR q < 0.05 were considered significant. And only several leading gene sets were displayed in the plot. (B) The enriched gene sets in HALLMARK by samples with low LRRC4C expression. (C) Enriched gene sets in C7 collection, the immunologic gene sets, by samples of high LRRC4C expression. Only several leading gene sets are shown in plot. (D) Enriched gene sets in C7 by the low LRRC4C expression.

**Figure 8:**
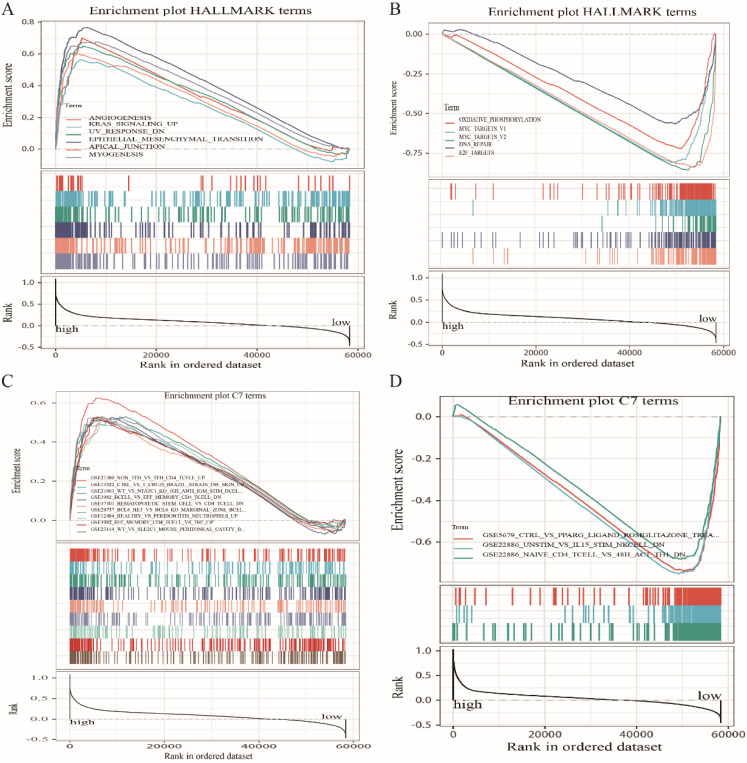
GSEA for COAD samples with high LRRC4C expression and low expression. (A) The enriched gene sets in HALLMARK collection by the high LRRC4C expression sample. Each line representing one particular gene set with unique color, and up-regulated genes located in the left approaching the origin of the coordinates, by contrast the down-regulated lay on the right of x-axis. Only gene sets with NOM p < 0.05 and FDR q < 0.05 were considered significant. And only several leading gene sets were displayed in the plot. (B) The enriched gene sets in HALLMARK by samples with low LRRC4C expression. (C) Enriched gene sets in C7 collection, the immunologic gene sets, by samples of high LRRC4C expression. Only several leading gene sets are shown in plot. (D) Enriched gene sets in C7 by the low LRRC4C expression.

Because *LRRC4C* expression negatively correlated with survival and TNM stages of STAD and COAD, we conducted GSEA and compared the results of the high- and low-expressing groups with median *LRRC4C* expression. Genes in the high *LRRC4C* expression group were mainly enriched in immune-related signalling pathways, including Hedgehog signalling, coagulation, KRAS signalling, and inflammatory responses (Figures 7A, 8A, and Table S2). Genes in the low *LRRC4C* expression group were mainly enriched in metabolic pathways, such as oxidative phosphorylation, and E2F targets (Figures 7A, 8A and Table S2). In the high-*LRRC4C*-expression group, multiple immune functional gene sets were enriched in the C7 collection defined by the Molecular Signatures Database (Figures 7C, 8C and Table S2). However, few gene sets were enriched in a group with low Bruton agammaglobulinemia tyrosine kinase expression (Figures 7D, 8D and Table S2). These results suggest *LRRC4C* as an indicator of TME immunity status in gastric and colon cancers.

### Correlation of *LRRC4C* with TIC proportion

**Figure 9:**
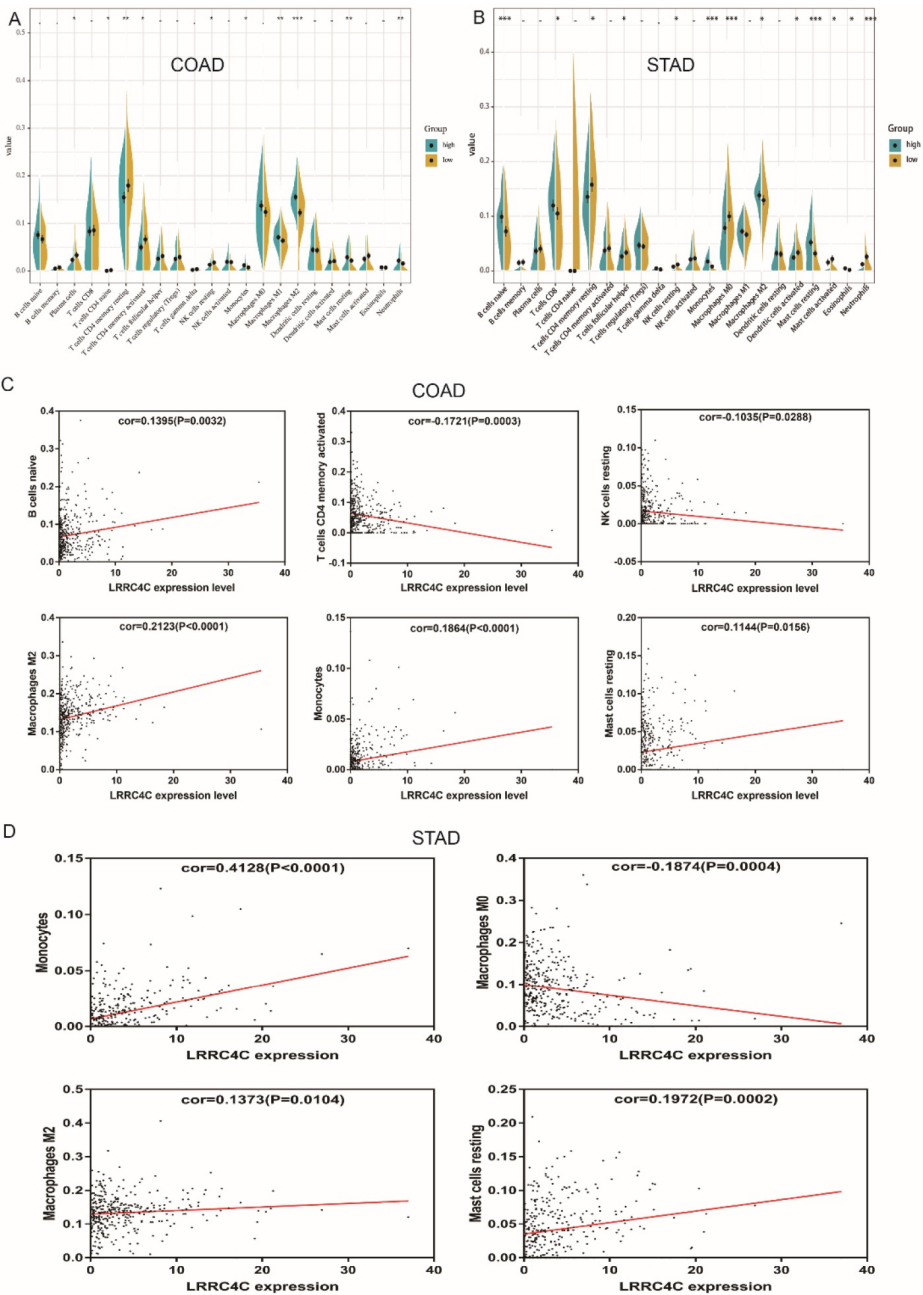
Correlation of TICs proportion with LRRC4C expression in COAD and STAD. (A, B) Violin plot showed the ratio differentiation of 22 kinds of immune cells between COAD or STAD samples with high or low LRRC4C expression relative to the median of LRRC4C expression level, and Wilcoxon rank sum was used for the significance test (*p<0.05, **p<0.01, ***p<0.001). (C) Scatter plot showed the correlation of 6 kinds of TICs proportion in COAD with the LRRC4C expression (p < 0.05). The red line in each plot was fitted linear model indicating the proportion tropism of the immune cell along with LRRC4C expression, and Pearson coefficient was used for the correlation test. (D) Scatter plot showed the correlation of 4 kinds of TICs proportion in STAD with the LRRC4C expression (p < 0.05).

The proportion of tumour-infiltrating immune subsets was analysed using the CIBERSORT algorithm, and 22 immune cell profiles in COAD and STAD samples were constructed. The results of difference and correlation analyses demonstrated that three types of TICs (monocytes, resting mast cells, and M2 macrophages) positively correlated with *LRRC4C* expression (Figure 9). These results further support the notion that *LRRC4C* levels affect the immune activity of the TME.

## Discussion

The present study found that immune and stromal scores of COAD samples from TCGA significantly correlated with clinical characteristics closely related to cancer prognosis (such as M stage, nerve invasion, and pathological type) and survival. These findings are similar to those previously obtained for gastric cancer 25. We attempted to identify TME-related genes that contributed to the survival and the clinical characteristics of patients with COAD. We demonstrated that LRRC4C is a prognostic marker of colon and gastric cancer and is involved in immune-related activities. More importantly, LRRC4C may participate along with monocytes, M2 macrophages, and resting mast cells in the construction of the TME in patients with colon and gastric cancers.

The TME plays a crucial role in the initiation and progression of tumorigenesis. Therefore, exploring potential therapeutic targets for TME remodelling that promote TME transformation from tumour-friendly to tumour-suppressive would be beneficial. Recent pre-clinical and clinical studies in cancer immunotherapy have drawn attention to the importance of the immunological tumour microenvironment 26. The results of our transcriptome analysis of the TCGA database show that the immune components and stromal components of the TME contribute to prognosis in patients with COAD. Similar results have been obtained in patients with STAD 25. The stromal and immune components in TME positively correlate with COAD tumour progression, mainly perineural invasion and distant metastasis. This suggests that remodelling the TME hinders tumour progression and improves patient prognosis.

Therefore, immunotherapy has become a promising therapeutic option for patients with cancer over the past few decades, although room for improvement remains. Human antibodies directed against immune checkpoint proteins such as cytotoxic T lymphocyte antigen-4, programmed death-1, and programmed death-ligand 1 have been applied to break immune tolerance and to stimulate T cell responses 27-29. Immune checkpoint inhibitors have elicited prolonged high response rates in subsets of patients with melanoma 30-32, renal 33, 34, and lung 35-37 cancers. However, clinical benefits of immunotherapy have not yet been bestowed upon patients with colon and gastric cancers, and a wide variety of immune-related adverse events cannot be ignored 38, and hence, novel candidates for immunotherapy against COAD and STAD require investigation. We applied ESTIMATE and CIBERSORT computational methods to analyse transcriptional information about COAD and STAD in TCGA and revealed the significant association of *LRRC4C* expression with advanced clinicopathological features (clinical stages and distant metastasis) and poor prognosis. These results were also verified by TIMER2.0, Kaplan-Meier-plotter, and GEPIA 2.0. Accordingly, LRRC4C might be a prognostic marker and therapeutic target for the TME in COAD and STAD.

The protein LRRC4C (also known as netrin-G1 ligand), belongs to a family of postsynaptic adhesion molecules that also includes netrin-G2 ligand/LRRC4, and netrin-G3 ligand/LRRC4B 13, 39. These NGLs are mainly expressed in the brain, although LRRC4C and LRRC4B mRNA has also been detected in the liver and heart, respectively 13, 39. As another member of the LRR superfamily, LRRC4 has the same intracellular structure as LRRC4C. It has been shown to plays an important role in neural development and malignant transformation of glioma, and has been identified as a tumour suppressor gene for glioma 14. Especially in recent years, there have been experiments to prove that LRRC4 is involved in nasopharyngeal carcinoma, pituitary adenoma and epithelial ovarian cancer 15, 40, 41, and more importantly, a certain common germline variant in LRRC4C has been implicated in confer increased risk of developing tumors in the central nervous system during childhood 42. Combined with our results, we have more reason to believe that LRRC4C is involved in the tumour progression of colon cancer and gastric cancer in a similar way to LRRC4. However, a relationship between LRRC4C and tumour development and metastasis had remained obscure. Therefore, we further analysed the relationship between *LRRC4C* expression and TME in COAD and STAD samples. The GSEA results showed that immune-related signalling pathways, such as Hedgehog, coagulation, KRAS signalling, and inflammatory responses, were significantly enriched in patients with high levels of *LRRC4C* expression. Metabolic pathways including oxidative phosphorylation, E2F targets, and Myc target V1 were enriched in samples expressing low levels of *LRRC4C*. These results imply that LRRC4C participates in the conversion of TME from immune-dominant to metabolic-dominant. LRRC4C might correlate with cell adhesion molecule binding and cell-cell adhesion mediator activity 43. This indicates that the expression of *LRRC4C* may be related to cell migration in the TME, thus leading to a disrupted balance of immunity. Further analysis of TIC supports this view. Accordingly, the upregulation of Bruton agammaglobulinemia tyrosine kinase along with advancing stages of lung adenocarcinoma, the conversion of TME from metabolic-dominant to immune escape status, and the addition of tumour promoting TICs support the notion that Bruton agammaglobulinemia tyrosine kinase plays an oncogenic role in COAD and STAD.

The relationship between *LRRC4C* expression and immune cells is unclear. Our CIBERSORT analysis of the proportion of TICs reveals that monocytes, resting mast cells, and M2 macrophages positively correlate with *LRRC4C* expression in patients with COAD and STAD. Monocytes can exert various functions at different stages of tumour growth and progression 44. Mast cell infiltration into tumours might remodel the TME and profoundly influence tumour behaviour by regulating and participating in inflammatory and immune reactions 45. M2 macrophages expressing the surface markers CD206 and CD204 promote the invasion and migration of gastric cancer cells by stimulating VEGF and MMP9 expression in cancer cells 46. Accumulating evidence indicates that tumour-associated macrophages contribute to angiogenesis, tumour growth, tumour progression, and metastasis 47. Therefore, the positive correlation between the amount of monocytes, resting mast cells, and M2 macrophages and *LRRC4C* expression in patients with COAD and STAD suggests that LRRC4C is responsible for preserving immune-active status in the TME.

In conclusion, we used the ESTIMATE algorithm to screen TCGA for TME-related genes in COAD samples and conducted functional enrichment and survival analyses. We found that LRRC4C is a potential prognostic factor for patients with COAD and STAD and may be an indicator of TME conversion from metabolic-dominant to immune escape. Therefore, further investigations clarifying the relationship between LRRC4C and monocytes, resting mast cells, and M2 macrophages are required to determine the promotion of colon cancer progression by LRRC4C and to stimulate new ideas for targeted therapy.

## Supplementary Material

Supplementary figures and tables.
