# Supplementary material for: Prognostic value of LRRC4C in Colon and Gastric Cancers correlates with Tumour Microenvironment Immunity
Source: Int J Biol Sci. 2021 Apr 3;17(5):1413–27. doi: 10.7150/ijbs.58876 (PMC8040466; doi:10.7150/ijbs.58876)
Supplement: Supplementary file 1 — Supplementary figures and tables. [file ijbsv17p1413s1.pdf]

**Supplementary Materials**

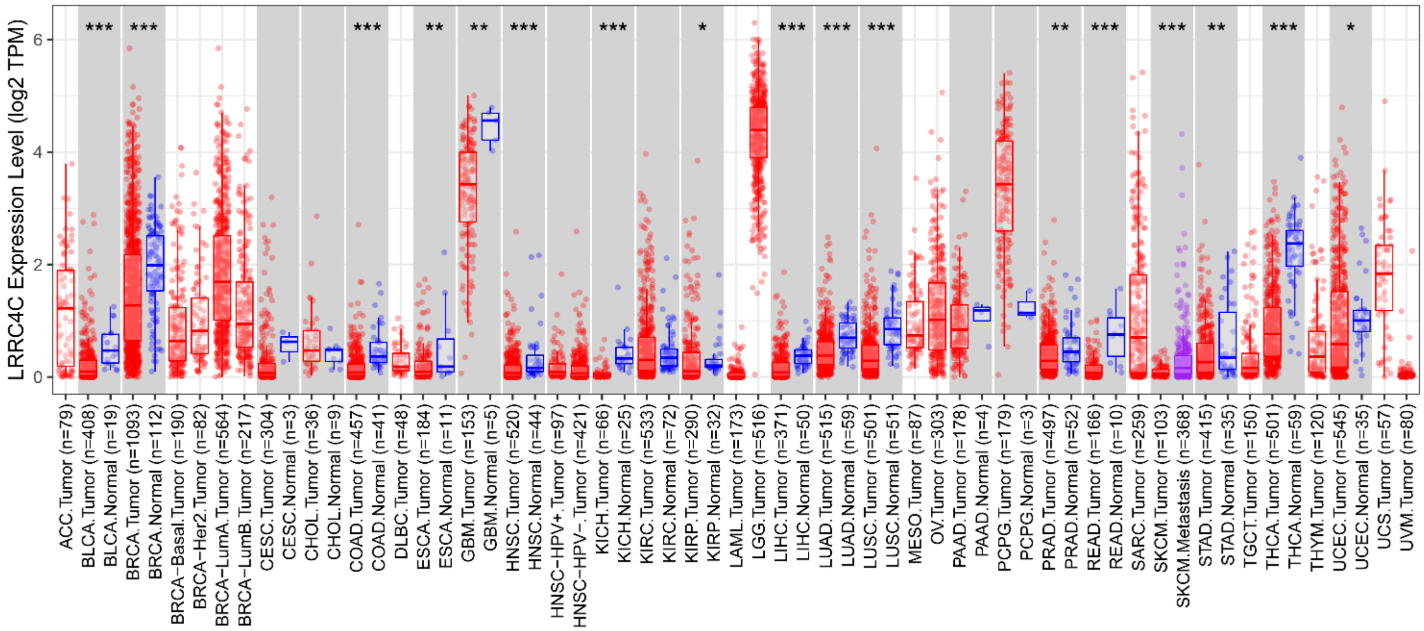


**Figure S1**. LRRC4C expression of different tumor types from the TCGA database was investigated by TIMER 2.0, mainly decreased expression of LRRC4C in different tumors compared to normal tissues (*P < 0.05, **P < 0.01, ***P < 0.001).

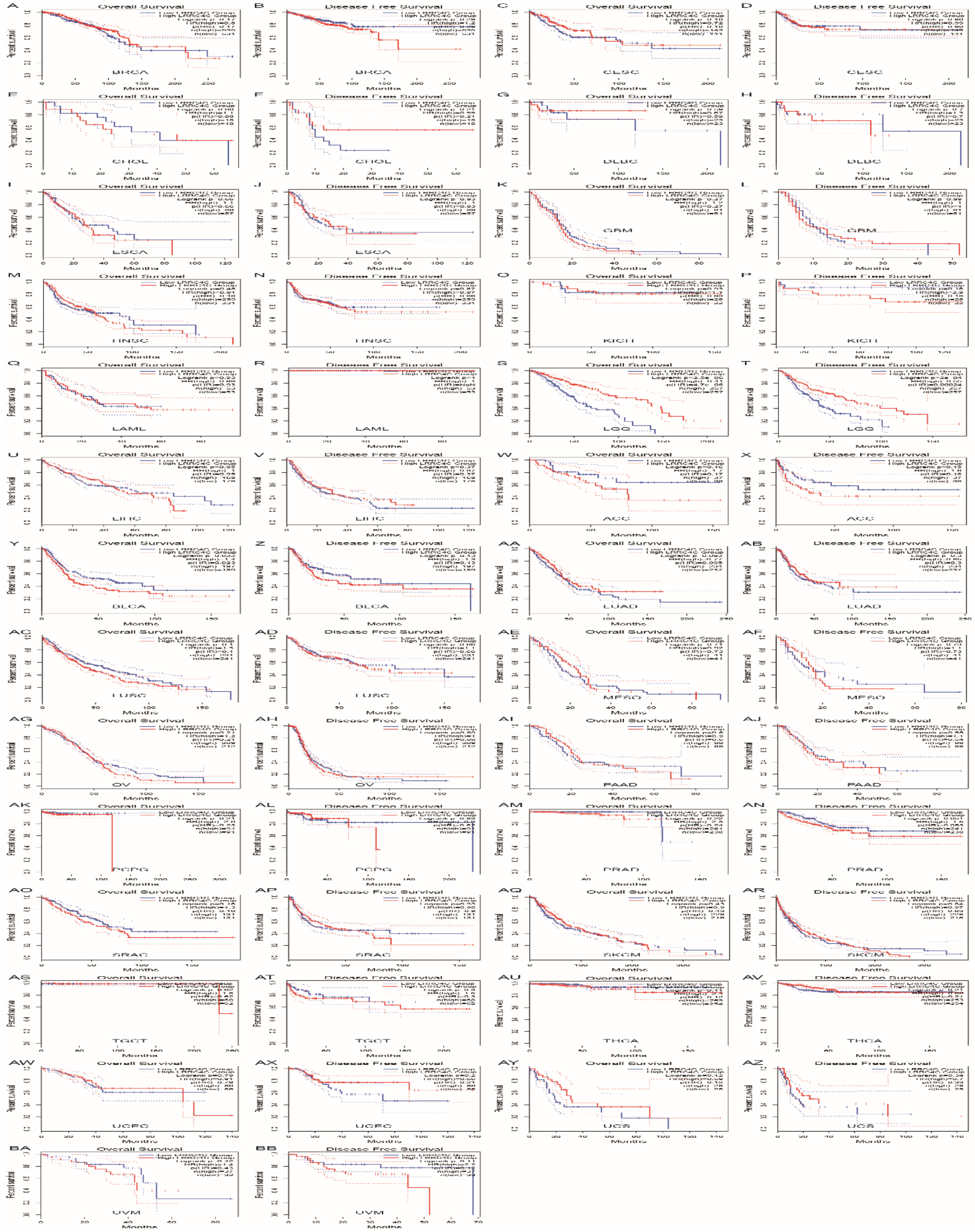


**Figure S2**. Correlation of LRRC4C expression with prognosis in diverse types of cancer in TCGA.

**Supplementary Table 1**. Univariate COX regression analysis with 58 DEGs among colon cancer patients from TCGA and GSE39582, listing the significant factors with $p < 0.05$.

| DEGs | HR | 95%CI lower | 95%CI upper | P-value |
|---|---|---|---|---|
| **COAD-OS** | | | | |
| ***LRRC4C*** | ***1.09*** | ***1.01*** | ***1.18*** | ***0.006*** |
| HAMP | 1.04 | 1.00 | 1.09 | 0.023 |
| **GSE39582-OS** | | | | |
| ***LRRC4C*** | ***3.71*** | ***2.45*** | ***4.96*** | ***2.14e-04*** |
| MCEMP1 | 1.38 | 1.07 | 1.69 | 3.78e-04 |
| CR1 | 1.91 | 1.29 | 2.53 | 0.001 |
| SEMA3D | 4.18 | 2.81 | 5.55 | 0.002 |
| CYP1B1 | 1.20 | 1.02 | 1.37 | 0.002 |
| LINC01094 | 1.31 | 1.05 | 1.56 | 0.005 |
| KCNJ15 | 1.40 | 1.08 | 1.73 | 0.006 |
| STAC | 1.69 | 1.19 | 2.20 | 0.006 |
| MARCO | 1.35 | 1.06 | 1.64 | 0.008 |
| FCN1 | 1.27 | 1.04 | 1.50 | 0.010 |
| SV2B | 2.20 | 1.44 | 2.95 | 0.015 |
| DCLK1 | 1.39 | 1.07 | 1.70 | 0.016 |
| LILRA2 | 1.70 | 1.19 | 2.21 | 0.018 |
| FMO2 | 1.45 | 1.09 | 1.81 | 0.022 |
| HS3ST2 | 1.35 | 1.06 | 1.63 | 0.034 |
| GAS1 | 1.15 | 1.02 | 1.29 | 0.034 |
| PPP2R2B | 2.08 | 1.38 | 2.79 | 0.035 |
| TREM1 | 1.13 | 1.01 | 1.25 | 0.038 |
| CNTN1 | 1.76 | 1.22 | 2.30 | 0.044 |
| **GSE39582-DFS** | | | | |
| SEMA3D | 6.12 | 4.38 | 7.85 | 2.26e-05 |
| MARCO | 1.58 | 1.14 | 2.02 | 4.37e-04 |
| MCEMP1 | 1.39 | 1.07 | 1.70 | 0.002 |
| KCND2 | 2.21 | 1.45 | 2.98 | 0.003 |
| STAC | 1.83 | 1.25 | 2.41 | 0.008 |
| ***LRRC4C*** | ***2.70*** | ***1.75*** | ***3.66*** | ***0.016*** |
| APCDD1L | 1.91 | 1.29 | 2.53 | 0.027 |
| LILRA2 | 1.72 | 1.20 | 2.23 | 0.038 |
| ABCA6 | 1.34 | 1.06 | 1.63 | 0.043 |
| FCN1 | 1.25 | 1.04 | 1.47 | 0.044 |

HR: hazard ratio; CI: confidence interval; DEGs: differentially expressed genes; OS: overall survival; DFS: disease free survival. COAD: colon adenocarcinoma, STAD: stomach adenocarcinoma.

**Supplementary Table 2**. GSEA results for COAD and STAD samples with high LRRC4C expression and low expression, only gene sets with NOM p < 0.05 and FDR q < 0.08 were considered significant, listing the top 15 significant gene sets.

| Name | NOM p-value | FDR q-value |
|---|---|---|
| **LRRC4C high expression-COAD-HALLMARK** | | |
| HALLMARK_MYOGENESIS | <0.0001 | 0.0042 |
| HALLMARK_EPITHELIAL_MESENCHYMAL_TRANSITION | 0.0020 | 0.0024 |
| HALLMARK_KRAS_SIGNALING_UP | <0.0001 | 0.0016 |
| HALLMARK_ANGIOGENESIS | <0.0001 | 0.0028 |
| HALLMARK_UCV_RESPONSE_DN | 0.0020 | 0.0026 |
| HALLMARK_APICAL_JUNCTION | 0.0040 | 0.0056 |
| HALLMARK_INFLAMMATORY_RESPONSE | 0.0078 | 0.0063 |
| HALLMARK_COAGULATION | 0.0020 | 0.0061 |
| HALLMARK_HEDGEHOG_SIGNALING | 0.0120 | 0.0142 |
| HALLMARK_COMPLEMENT | 0.0172 | 0.0139 |
| HALLMARK_IL2_STAT5_SIGNALING | 0.0174 | 0.0190 |
| HALLMARK_APICAL_SURFACE | 0.0136 | 0.0423 |
| HALLMARK_IL6_JAK_STAT3_SIGNALING | 0.0470 | 0.0400 |
| HALLMACRK_ALLOGRAFT_REJECTION | 0.0434 | 0.0407 |
| HALLMARK_KRAS_SIGNALING_DN | 0.0108 | 0.0467 |
| **LRRC4C low expression-COAD-HALLMARK** | | |
| HALLMARK_OXIDATIVE_PHOSPHORYLATION | <0.0001 | 0.0338 |
| HALLMARK_E2F_TARGETS | 0.0062 | 0.0233 |
| HALLMARK_MYC_TARGETS_V1 | <0.0001 | 0.0496 |
| HALLMARK_MYC_TARGETS_V2 | <0.0001 | 0.0485 |
| HALLMARK_PEROXISOME | 0.0144 | 0.0405 |
| HALLMARK_DNA_REPAIR | 0.0230 | 0.0402 |
| **LRRC4C high expression-COAD-C7** | | |
| GSE21063_WT_VS_NFATC1_KO_16H_ANTI_IGM_STIM_BCELL_DN | <0.0001 | 0.0010 |
| GSE28737_BCL6_HET_VS_BCL6_KO_MARGINAL_ZONE_BCELL_UP | <0.0001 | 0.0011 |
| GSE3982_BCELL_VS_EFF_MEMORY_CD4_TCELL_DN | <0.0001 | 0.0021 |
| GSE21380_NON_TFH_VS_TFH_CD4_TCELL_UP | <0.0001 | 0.0053 |
| GSE23114_WT_VS_SLE2C1_MOUSE_PERITONEAL_CAVITY_B1A_BCELL_DN | <0.0001 | 0.0084 |
| GSE13522_CTRL_VS_T_CRUZI_BRAZIL_STRAIN_INF_SKIN_UP | <0.0001 | 0.0093 |
| GSE3982_EFF_MEMORY_CD4_TCELL_VS_TH1_UP | <0.0001 | 0.0094 |
| GSE37301_HEMATOPOIETIC_STEM_CELL_VS_CD4_TCELL_DN | <0.0001 | 0.0107 |
| GSE27786_NKTCELL_VS_ERYTHROBLAST_DN | <0.0001 | 0.0133 |
| GSE19825_NAIVE_VS_IL2RAHIGH_DAY3_EFF_CD8_TCELL_UP | <0.0001 | <0.0001 |
| GSE1791_CTRL_VS_NEUROMEDINU_IN_T_CELL_LINE_0.8H_DN | <0.0001 | <0.0001 |
| GSE19825_NAIVE_VS_IL2RALOW_DAY3_EFF_CD8_TCELL_UP | <0.0001 | <0.0001 |
| GSE23502_BM_VS_COLON_TUMOR_HDC_KO_MYELOID_DERIVED_SUPPRESSOR_CELL_UP | <0.0001 | <0.0001 |
| GSE13411_NAIVE_VS_MEMORY_BCELL_UP | <0.0001 | <0.0001 |

| Name | NOM p-value | FDR q-value |
|---|---|---|
| **LRRC4C low expression-COAD-C7** | | |
| GSE22886_NAIVE_CD4_TCELL_VS_48H_ACT_TH1_DN | 0.0490 | 0.0250 |
| **LRRC4C high expression-STAD-HALLMARK** | | |
| HALLMARK_MYOGENESIS | <0.0001 | 0.0188 |
| HALLMARK_APICAL_JUNCTION | 0.0039 | 0.0537 |
| HALLMARK_EPITHELIAL_MESENCHYMAL_TRANSITION | 0.0131 | 0.0506 |
| HALLMARK_UV_RESPONSE_DN | 0.0083 | 0.0545 |
| HALLMARK_KRAS_SIGNALING_UP | 0.0179 | 0.0652 |
| HALLMARK_ANGIOGENESIS | 0.0094 | 0.0575 |
| HALLMARK_COAGULATION | 0.0191 | 0.0780 |
| HALLMARK_KRAS_SIGNALING_DN | 0.0020 | 0.0772 |
| HALLMARK_HEDGEHOG_SIGNALING | 0.0242 | 0.0768 |
| HALLMARK_IL2_STAT5_SIGNALING | 0.0433 | 0.0718 |
| **LRRC4C low expression-STAD-HALLMARK** | | |
| HALLMARK_OXIDATIVE_PHOSPHORYLATION | 0.0020 | 0.0014 |
| HALLMARK_MYC_TARGETS_V1 | <0.0001 | 0.00333 |
| HALLMARK_MYC_TARGETS_V2 | <0.0001 | 0.0129 |
| HALLMARK_DNA_REPAIR | 0.01 | 0.0135 |
| HALLMARK_E2F_TARGETS | <0.0001 | 0.0123 |
| HALLMARK_UNFOLDED_PROTEIN_RESPONSE | 0.0175 | 0.0120 |
| HALLMARK_MTORC1_SIGNALING | 0.0101 | 0.0104 |
| HALLMARK_G2M_CHECKPOINT | 0.0020 | 0.0119 |
| HALLMARK_FATTY_ACID_METABOLISM | 0.0238 | 0.0473 |
| HALLMARK_GLYCOLYSIS | 0.0178 | 0.0436 |
| HALLMARK_PEROXISOME | 0.0215 | 0.0678 |
| HALLMARK_CHOLESTEROL_HOMEOSTASIS | 0.0478 | 0.0793 |
| **LRRC4C high expression-STAD-C7** | | |
| GSE23114_WT_VS_SLE2C1_MOUSE_PERITONEAL_CAVITY_B1A_BCELL_DN | <0.0001 | 0.0500 |
| GSE12484_HEALTHY_VS_PERIDONTITIS_NEUTROPHILS_UP | <0.0001 | 0.0499 |
| GSE28737_BCL6_HET_VS_BCL6_KO_MARGINAL_ZONE_BCELL_UP | <0.0001 | 0.0498 |
| GSE37301_HEMATOPOIETIC_STEM_CELL_VS_CD4_TCELL_DN | <0.0001 | 0.0497 |
| GSE3982_BCELL_VS_EFF_MEMORY_CD4_TCELL_DN | <0.0001 | 0.0497 |
| GSE21063_WT_VS_NFATC1_KO_16H_ANTI_IGM_STIM_BCELL_DN | <0.0001 | 0.04945928 |
| GSE13522_CTRL_VS_T_CRUZI_BRAZIL_STRAIN_INF_SKIN_UP | <0.0001 | 0.0494 |
| GSE21380_NON_TFH_VS_TFH_CD4_TCELL_UP | <0.0001 | 0.0485 |
| GSE27786_NKTCELL_VS_ERYTHROBLAST_DN | <0.0001 | 0.0490 |
| GSE28783_CTRL_ANTI_MIR_VS_UNTREATED_ATHEROSCLEROSIS_MACROPHAGE_UP | <0.0001 | 0.0423 |
| GSE37301_CD4_TCELL_VS_RAG2_KO_NK_CELL_UP | <0.0001 | 0.0423 |
| GSE21546_UNSTIM_VS_ANTI_CD3_STIM_DP_THYMOCYTES_UP | <0.0001 | 0.0423 |
| GSE43863_NAIVE_VS_LY6C_INT_CXCR5POS_CD4_EFF_TCELL_D6_LCMV_DN | <0.0001 | 0.0423 |
| GSE29949_MICROGLIA_BRAIN_VS_MONOCYTE_BONE_MARROW_UP | 0.0021 | 0.0423 |

| Name | NOM p-value | FDR q-value |
|---|---|---|
| GSE13485_DAY3_VS_DAY21_YF17D_VACCINE_PBMC_DN | <0.0001 | 0.0423 |
| GSE10325_LUPUS_CD4_TCELL_VS_LUPUS_BCELL_UP | 0.0199 | 0.0423 |
| **LRRC4C low expression-STAD-C7** | | |
| GSE15930_NAIVE_VS_48H_IN_VITRO_STIM_CD8_TCELL_DN | 0.0199 | 0.007 |
| GSE21360_SECONDARY_VS_TERTIARY_MEMORY_CD8_TCELL_UP | 0.0197 | 0.017 |
| GSE15930_NAIVE_VS_48H_IN_VITRO_STIM_IFNAB_CD8_TCELL_DN | 0.0110 | 0.019 |
| GSE41867_DAY6_VS_DAY8_LCMV_ARMSTRONG_EFFECTOR_CD8_TCELL_DN | 0.0140 | 0.019 |
| GSE22886_NAIVE_CD4_TCELL_VS_48H_ACT_TH1_DN | 0.0086 | 0.044 |

GSEA: Gene Set Enrichment Analysis, COAD: colon adenocarcinoma, STAD: stomach adenocarcinoma.
